# Self-Rotation of Cells in an Irrotational AC E-Field in an Opto-Electrokinetics Chip

**DOI:** 10.1371/journal.pone.0051577

**Published:** 2013-01-08

**Authors:** Long-Ho Chau, Wenfeng Liang, Florence Wing Ki Cheung, Wing Keung Liu, Wen Jung Li, Shih-Chi Chen, Gwo-Bin Lee

**Affiliations:** 1 Centre for Micro and Nano Systems, The Chinese University of Hong Kong, Hong Kong; 2 State Key Laboratory of Robotics, Shenyang Institute of Automation, Chinese Academy of Sciences, Shenyang, China; 3 School of Biomedical Sciences, Faculty of Medicine, The Chinese University of Hong Kong, Hong Kong; 4 Department of Mechanical and Biomedical Engineering, City University of Hong Kong, Hong Kong; 5 Department of Power Mechanical Engineering, National Tsing Hua University, Hsinchu, Taiwan; Queen's University at Kingston, Canada

## Abstract

The use of optical dielectrophoresis (ODEP) to manipulate microparticles and biological cells has become increasingly popular due to its tremendous flexibility in providing reconfigurable electrode patterns and flow channels. ODEP enables the parallel and free manipulation of small particles on a photoconductive surface on which light is projected, thus eliminating the need for complex electrode design and fabrication processes. In this paper, we demonstrate that mouse cells comprising melan-a cells, RAW 267.4 macrophage cells, peripheral white blood cells and lymphocytes, can be manipulated in an opto-electrokinetics (OEK) device with appropriate DEP parameters. Our OEK device generates a non-rotating electric field and exerts a localized DEP force on optical electrodes. Hitherto, we are the first group to report that among all the cells investigated, melan-a cells, lymphocytes and white blood cells were found to undergo self-rotation in the device in the presence of a DEP force. The rotational speed of the cells depended on the voltage and frequency applied and the cells' distance from the optical center. We discuss a possible mechanism for explaining this new observation of induced self-rotation based on the physical properties of cells. We believe that this rotation phenomenon can be used to identify cell type and to elucidate the dielectric and physical properties of cells.

## Introduction

Single-cell manipulation plays an important role in the biomedical research fields of culturing, drug development, physiology and replication. Existing methods for manipulating a single biological cell are applied in microfluidic devices. The successful application of mechanical based techniques such as hydrodynamic flow [1]–[2], laminar flow control [3]–[4] and micromechanical filters [5] have been successfully demonstrated in cell transportation and separation by considering the size and morphology of cells. The channel fabricated while applying these techniques is exclusively designed for particles and cells of a specific size and provides them with a desired path to move along. However, the device may not perform properly if larger or smaller particles are injected into the system for transportation or separation. In many such cases, engineers must redesign the channel and micro-fabricate the device again. Electrokinetic based methods including (AC) electro-osmosis (EO) [6], electrophoresis (EP) [7], dielectrophoresis (DEP) [8]–[10] and isoelectrophoresis (IEP) [11] can precisely transport bulky cells to a desired position according to their size and dielectric properties. In all such methods, chips with patterned electrodes are required to guide the cells in the specified direction. These methods, however, cannot be employed to manipulate a single cell without an appropriately designed channel. They also require complex fabrication processes such as metal deposition, lithography and channel fabrication. Moreover, although they guide cells along a specific track, they cannot manipulate them into other positions.

An enhanced DEP-based technology called optical dielectrophoresis (ODEP) has recently been developed to manipulate cells and particles using focused light and an AC E-field [12]–[14]. Under ODEP, an opto-electrokinetics (OEK) device is used to perform cell/particle manipulation. The device consists of a smooth photoconductive layer and a conductive chip. A localized DEP force is generated across the OEK device when an optical image emitted from a light source, such as a laser or projector, is projected onto the photoconductive layer. Because the position of the DEP force varies with the location of the image, the optical image acts as a cell manipulation controller. Hence, cells can move in any direction within a void volume with sufficient DEP force.

ODEP has been used to successfully manipulate nano-/micro- particles/biological cells. The literature has shown that the general motion of particles in an OEK device is purely translational [13]–[15], with the E-field generated being irrotational. However, according to YL Liang et al. (2011), yeast cells undergo translation and rotation at the same time from the dark-field region to the image spot [16]. The same study also demonstrated Ramos cells generating self-rotation in an OEK device with the aid of a well. Both rotation observations were performed in a rotating E-field. In this paper, we present our recent findings on cell rotation among some of the cells examined in an irrotational E-field using an OEK device. White blood cells (WBCs) and melan-a cells were observed to undergo self-rotation near the image spot at an appropriate applied frequency and voltage. Neither physical barriers nor other mechanical barriers, such as a well, were applied to assist the rotation of the cells.

## Theory

**Figure 1:**
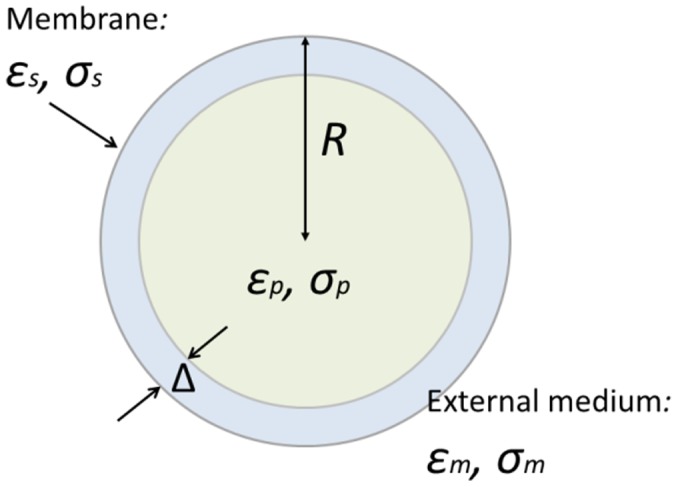
The single-shell model. A spherical particle with radius *R*, permittivity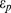 and conductivity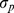, which is covered by a uniform layer of thickness 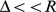, permittivity 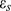 and conductivity 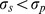, and surrounded by a solution of permittivity 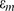 and conductivity 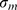.

An ODEP system consists of optics and DEP components. Optics, i.e. projected light or images, control the position of the DEP force in an OEK device. When there is no optical image (light), no AC E-field is generated across the device. When light is projected onto a particular area of the photoconductive layer, a localized DEP field is generated across the illuminated area and the conductive layer. The average time it takes for a DEP force to act on a round cell in an OEK device is expressed in Equation (1) with the assumption that the cell suspended in the medium is perfectly spherical [17]:
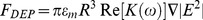
(1)where 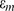 is the dielectric permittivity of the medium, *R* is the radius of the particle, 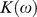 is the Clausius-Mossotti (CM) factor, 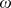 is the applied angular frequency across the medium and *E* is the electric field. An animal cell consists of a nucleus, cytoplasm and other organelles and is surrounded by a membrane. It can be considered a single-shell model rather than a homogeneous sphere, as shown in Figure 1. The CM factor is therefore expressed as [18]:
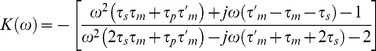
(2)where 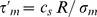, 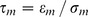, 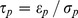 and 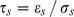. 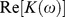 ranges from −0.5 to 1 and its sign determines the direction of the DEP force. If 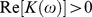, a positive DEP force is generated in such a way that the particles/cells tend to move toward a strong E-field region. If 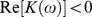, a negative DEP is generated, and the particles are thus pushed toward a weak E-field region.

Several studies in the literature have demonstrated that particular cells can rotate in a two-phase-shifted DEP E-field and in a travelling wave DEP due to electrorotation. The real part of Equation (2) determines the direction of the DEP force while the imaginary part determines the electrorotation of the particles in the rotating E-field. DEP theory states that the action of an externally applied E-field on a polarizable particle results in the formation of an induced dipole moment. When a dipole sits in a uniform E-field, each charge on the dipole is parallel to the field; hence, it experiences a torque. If the direction of the field vector changes, the induced dipole moment vector must realign itself with the E-field vector, which causes particle rotation to occur. The torque of the particle in a rotating E-field [17] is expressed as
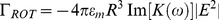
(3)Electrorotation occurs if the E-field has a spatially dependent phase, otherwise 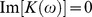. Dielectrophoresis and electrorotation are tools commonly applied to measure the dielectric properties of biological cells [19]. The data obtained can be used to identify different cell types or to form a cell library. Scientists and engineers often use these electrical parameters to manipulate cells, such as in cell separation and transportation using DEP or ODEP.

In this study, our research group observed the self-induced rotation of melan-a, lymphocytes and WBCs in an ODEP system. The cells could rotate during stationary and translational motion in a non-uniform E-field. In a notable finding, the AC E-field generated in the OEK device did not rotate to cause electrorotation; hence, no torque was generated according to Equation (3).

## Materials and Methods

### Instrumentation

**Figure 2:**
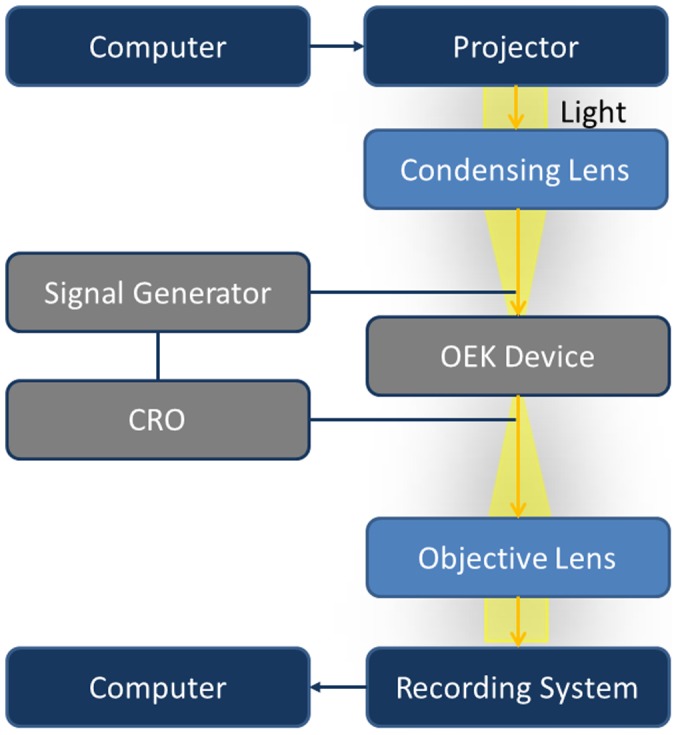
Illustration of an ODEP system. Experimental setup for manipulating cells with opto-electrokinetic device.

The ODEP system employed in this study is shown in Figure 2 and Figure S1. The OEK chip was placed on a 2D stage integrated with an optical microscope (Nikon ECLIPSE TE2000-U). Light was projected from a commercial projector (DELL 1510X) and passed through the condensing lens (Leica X/0.15), indium tin oxide (ITO) glass and a liquid medium before being focused on the hydrogenated amorphous silicon (a-Si:H) surface. Image patterns of light were controlled by a computer. Cell motion was recorded using a high-speed camera (PCO 1200S) with a 40× objective lens. The top and bottom ITO glass chips were connected to an AC signal generator and a CRO. In each experiment, a drop of cell solution was injected into the OEK device and there was no net fluid flow in the micro-chamber. The frequencies applied for cell manipulation ranged from 10 kHz to 3 MHz and the voltages applied were from 0 V to 20 V, peak to peak. All experiments were performed at room temperature.

### Chemical and cell preparation

Immortalized mouse melanocyte, melan-a pigment cells (a gift from Prof. D.C. Bennett at St. George's Hospital Medical School in London, UK) [20] and non-pigment cells, including the mouse RAW 264.7 macrophage cell line (ATCC TIB-71), mouse peripheral white blood cells and lymphocytes were suspended in 0.2 M sucrose in deionized water. The measured conductivity of the sucrose was 0.37 mSm^−1^. The experiments were performed immediately after the fresh cells were prepared.

### Fabrication of opto-electrokinetics device

#### a) Fabrication of OEK photoconductive layer

The fabrication of the glass-ITO-a-Si:H structure employed in this study was described by Yen-Heng Lin et al. (2010). [14]A 0.3 µm a-Si:H thin film was coated on the ITO layer. It was further processed by etching part of the a-Si:H for electrical connection. A 5 mm×8 mm area of a-Si:H was patterned through standard photolithography and dry-etching using the Oxford Plasma Lab 80 Plasma Etching System with 2% oxygen, 12.5% CF_4_ gas, a 30 mTorr etching chamber and 6-minute plasma exposure. The chip was then rinsed and cleaned with acetone and DI water before being dried by nitrogen gas.

#### b) Fabrication of OEK device

**Figure 3:**
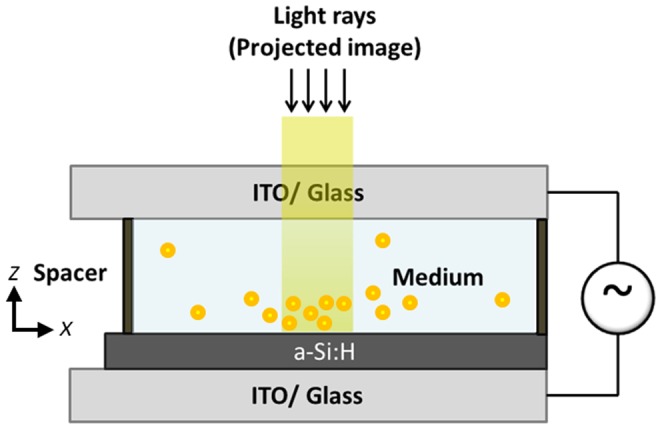
The opto-electrokinetics device (OEK). An illustration of the OEK used to manipulate biological cells. The patterned optical image is focused by a condenser lens and projected onto the hydrogenated amorphous silicon surface.

The OEK device was assembled by combining an amorphous silicon a-Si-coated glass and an ITO-coated glass with the SU-8 intermediate layer as shown in Figure 3. The SU-8 layer (SU-8 2035, MicroChem, Newton, MA, USA) served as a micro-fluidic channel for cell-medium transportation and cell separation. The ITO glass was prepared by sputtering 600 Å ITO on clean glass for 10 minutes. A few through-holes were then created in the ITO glass to serve as channel ports. The fabrication process for the SU-8 micro-fluidic channel consisted of several steps. First, an uncross-linked SU-8 negative photoresist was spun on an a-Si glass at 500 rpm for 10 seconds, 1000 rpm for 60 minutes and 5000 rpm for 3 seconds to obtain a thickness of 50 µm, which was followed by a two-step soft baking process on a hotplate at 65°C for 3 minutes, then at 95°C for 6 minutes. The soft-baked SU-8 was exposed to UV light using a Karl Suss MA4 mask aligner for 70 seconds. Subsequently, the exposed SU-8 layer was hard-baked following a two-step approach: it was initially baked at 65°C for 1 minute, then at 95°C for 5 minutes. The resulting SU-8 layer was cooled to room temperature and kept for 4 hours before being subjected to a developing process to strengthen the adhesion of the SU-8 and a-Si layers. In the final step, the cross-linked SU-8 was developed, rinsed with isopropanol (IPA) and dried by nitrogen gas.

The a-Si glass/SU-8 structure, thus prepared, was sealed to the ITO glass by employing the SU-8 imprinting method shown in Figure S2. First, a cleaned transparency was spin-coated with a 10 µm SU-8 2010 layer at 500 rpm for 10 seconds and 3000 rpm for 30 seconds. The patterned SU-8 structure was then imprinted on the uncross-linked SU-8 coated transparency to allow the bulge surface to make good contact with the SU-8. The transparency was then detached from the SU-8 structure. The structured SU-8/a-Si glass and the ITO glass were then aligned and brought into contact. They were then clamped tightly and baked in a 150°C oven for 30 minutes.

## Results and Discussion

### Investigation of cell properties and translation behavior under ODEP

#### a) WBCs

Peripheral white blood cells (leukocytes) collected from male adult C57 mice consist of different types of cells including lymphocytes, macrophages and polymorphonucelar cells, which can be categorized into two main types: granulocytes and agranulocytes. Granulocytes are white blood cells that contain granules in their cytoplasm; neutrophils, basophils and eosinophils belong to this group. Agranulocytes are white blood cells that do not contain granules, such as monocytes and lymphocytes. Here, we discuss the rotation and translation behavior of three kinds of cells under the ODEP system: white blood cells, lymphocytes and macrophages.

**Figure 4:**
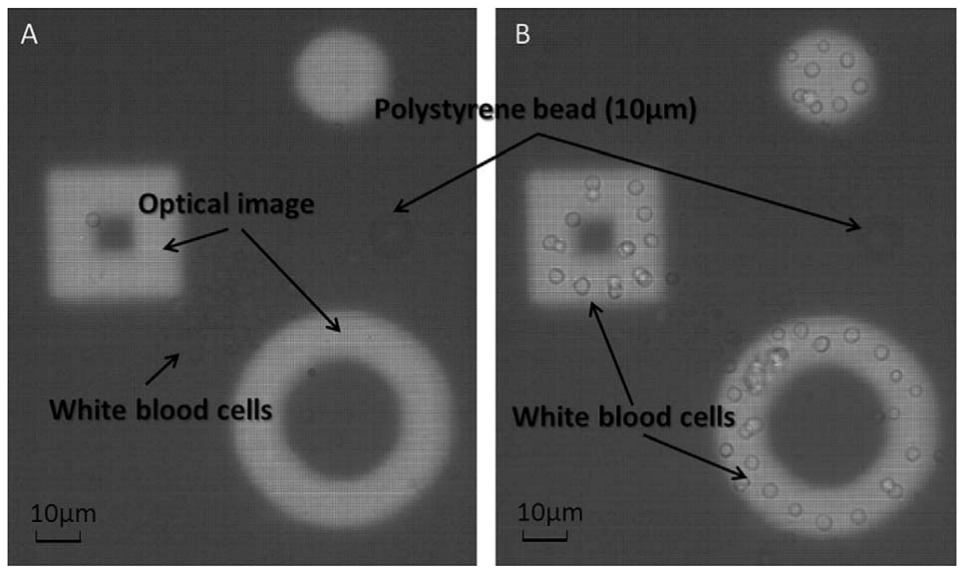
DEP force acting on the peripheral WBCs. Three optical images were projected onto an a-Si:H surface. A 10 µm diameter micro polystyrene bead acted as a control. (A) Peripheral WBCs experienced a negative DEP force at the applied frequency of 50 kHz and a voltage of 10 Vpp. They lay in the dark-field region and between the square and the ring image. (B) When the frequency was 200 kHz, 10 Vpp, a positive DEP force caused the cells to shift into the image. The polystyrene beads stayed in the same position in both cases because they only experienced a negative DEP force in 0.2 M sucrose solution.

Three primary mouse blood samples were collected from the abdominal aorta in saline solution, with or without heparin, and from the spleens of C57 mice. White blood cells were obtained after the lysis of red blood cells in ammonium chloride solution. WBC in saline without heparin (WBCs), with heparin (h-WBCs) and from the spleen (s-WBCs) were analyzed in an OEK device. Frequencies ranging from 5 kHz to 300 kHz at 20 Vpp were applied to the OEK chip and the results are shown in Movie S2 and summarized in Table 1. All samples experienced a negative DEP force when the frequency was less than 50 kHz. The cells moved away from the virtual image when a negative DEP force was applied, as shown in Figure 4. When the frequency was increased to 100 kHz, 50% of WBCs, 50% of h-WBCs and 30% of s-WBCs experienced a positive DEP force. As such, they were attracted from the dark-field region to the bright-field area (virtual image). As the cells translated from the dark-field region toward the image spot, they also generated rotational motion. The rotation axis was perpendicular to the E-field and the cells rotated toward the image. When the applied frequency reached 200 kHz, most of the WBCs and h-WBCs invaded the image spot by means of a positive DEP force. However, only half of the s-WBCs experienced a positive DEP force at 200 kHz and above, which indicated that half of the s-WBCs did not have a positive DEP frequency spectrum in the studied medium.

**Table 1:** The response of white blood cell samples in the DEP field.

Applied Frequency (Hz) in 20 Vpp		<50 kHz	100 kHz	200 kHz <
**DEP Force**	WBCs	Negative (100%)	Negative (50%) & Positive (50%)	Positive (90%)
	h-WBCs	Negative (100%)	Negative (50%) & Positive (50%)	Positive (90%)
	s-WBCs	Negative (100%)	Negative (70%) & Positive (30%)	Negative (50%) & Positive (50%)

#### b) Macrophages

Mouse macrophages from the RAW 264.7 cell line have previously been investigated by our group [21]. Their diameter is approximately 10 µm and they can only be manipulated by a negative DEP force in the 50 kHz to 150 kHz frequency range. When 10 Vpp and 150 kHz was applied to an OEK device, the macrophage left the image at a speed of 2 µm/s. We found that the macrophages did not self-rotate at any applied frequency or voltage.

#### c) Lymphocytes

Lymphocytes have a large nucleus that usually occupies 80% of the whole cell [22]. They have a spherical shape and range in size from 6 µm to 7 µm. We tested frequencies ranging from 25 kHz to 3 MHz. The lymphocytes experienced a negative DEP force below 40 kHz, causing them to repel from the optical image and rotate in the dark-field region. When the frequency was increased to between 50 kHz and 800 kHz, the cells were subjected to a positive DEP force and tended to move toward the optical image. However, they eventually stopped some distance from the image and underwent self-rotation in that position. For example, the cells shifted from 150 µm at the image spot to 65 µm away from the image spot if the frequency changed from 40 kHz to 60 kHz. We believe that there was a ‘repulsive force’ around the image boundary which is possibly generated by counter-rotating electrothermal flow structures around the image. This ‘repulsive force’ is apparently stronger than the attractive DEP force acting on the cells when the cells are close to the image, which causes the cells from further translation in to the image. At 140 kHz, the cells experienced the strongest DEP force; some cells moved inside the spot, whereas others stayed 30 µm from the spot. For frequencies above 800 kHz, the cells did not translate and/or rotate in any direction because there was insufficient DEP force acting on them.

#### d) Melan-a

Melan-a is an immortalized mouse melanocyte line. Melanocytes are melanin-producing cells located in the bottom layer (the stratum basale) of the skin's epidermis. Melanin is commonly found in most organisms. It determines the color of the skin, hair and iris. Melan-a cells vary in size from 8 µm to 10 µm and appear to have a sphere-like shape when suspended in a medium, although their membrane is not smooth. Our group has previously examined their translational motion under ODEP [21]. The responses of melan-a cells were found to be very similar to those of lymphocytes in that some melan-a cells did not shift into the image spot when a positive DEP force was applied to them, as shown in Movie S1. These cells stayed at a particular position around the image spot and underwent self-rotation; for instance, at 20 Vpp and 100 kHz, they rotated at 90 rpm and remained at a distance of 10 µm to 15 µm from the image spot. Table 2 summarizes the responses of the melan-a and macrophage cells in the OEK device.

**Table 2:** The response of melan-a cells and macrophages to the DEP field.

Applied Frequency (Hz) in 20 Vpp		<25 k	25 k–50 k	50 k–100 k	100 k–300 k	>300 k
**DEP Force**	Melan-a	Positive DEP	Positive DEP	Positive DEP	Positive DEP	No Response
		All cells were strongly attracted to the image and then burst.	80% of the cells were attracted to the image. Other cells stayed stationary near the image.	Some cells were attracted to the image and some cells stayed stationary near the image.	70% of the cells moved towards the image and then stayed stationary near the image.	No movement of the cells was observed.
	Macrophage	No Response	No Response	Weak negative DEP	Negative DEP	No Response
		No movement of the cells was observed.	No movement of the cells was observed.	Cells started to repel from the image slowly.	Cells moved away from the image at the speed of 2 µm/s at 150 kHz.	No movement of the cells was observed.

### Investigation of cellular self-rotation behavior

**Figure 5:**
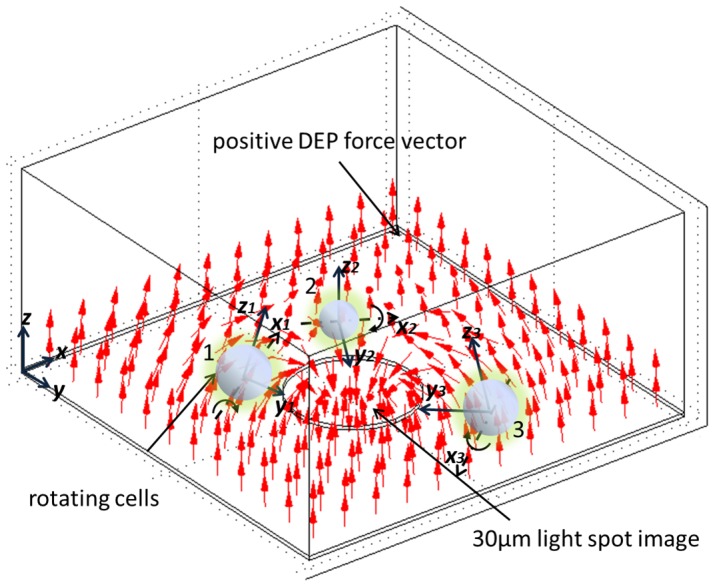
Illustration of cell rotation in an OEK. The cells in the dark-field region rotated toward the 30 µm spot image. Their *z*-axes were normal to the positive DEP force vectors. The axis of rotation was at the *x*-axis perpendicular to the E-field.

As previously described, lymphocytes and melan-a cells in the dark-field region were found to rotate near the image spot under both positive and negative DEP forces. The rotation axes for both cells were perpendicular to the applied E-field and the direction of rotation was toward the image spot, as shown in Figure 5. In this section, we analyze the relationship of cell rotational speed to applied voltage and frequency.

#### a) Rotation Measurement

**Figure 6:**
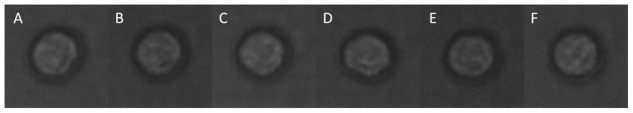
Rotation of melan-a. Time lapse for a melan-a cell completing one revolution with the applied frequency of 40 kHz at 6 Vpp as recorded by a CCD camera after grey-scale treatment. The similarity coefficients were 1 (A), 0.9973 (B), 0.9964 (C), 0.99656 (D), 0.9951(E) and 0.9963(F). Images were taken 0.155 seconds apart. The rotational speed was recorded as 65 rpm.

Cell motion was captured using a CCD camera with a fixed frame rate of 64.5/s. The images captured were processed by resizing the image and focusing on the cells as shown in Figure 6. The modified cell images were then imported into rotation tracking software developed by our group. The algorithm used for the tracking process first identifies the feature points of each imported cell image before calculating the length differences among the images in comparison with the first imported image and generating a mapping similarity index for the cell ranging from 0 to 1 (with 1 meaning identical and 0 meaning not correlated). For example, the first imported image was assigned an index of 1 as a reference, the second image was calculated a value of 0.9973 because its orientation differs from that of the first image, the third image was calculated a value of 0.9964 and for a complete cell rotation loop, its index was 0.9963, compared with the first image. We can eventually identify the rotation period by looking at the similarity index between sequences and calculating the length of the frames.

#### b) Rotational Speed vs. Voltage

**Figure 7:**
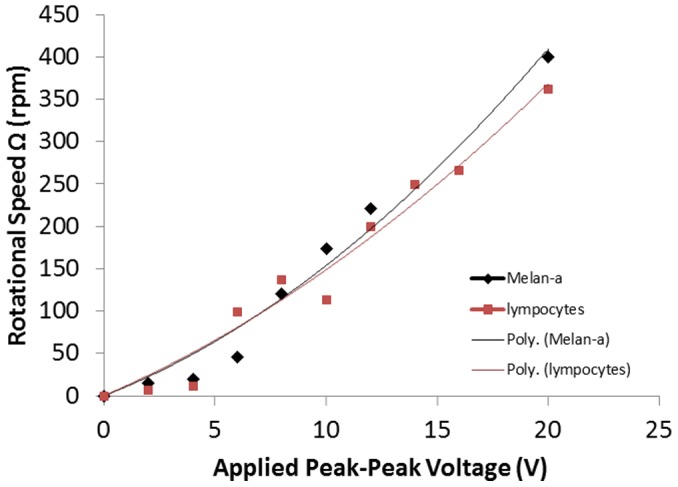
Rotational speed of the melan-a and lymphocytes in 0.2 M sucrose solution versus applied voltage from 0 Vpp to 20 Vpp at 40 kHz.

Figure 7 shows the rotational speed of melan-a cells at applied voltages from 0 Vpp to 20 Vpp at 40 kHz. The melan-a cells located 65 µm from the light spot were recorded. The cells were originally at rest from 0 Vpp to 1 Vpp. They started self-rotating at 15 rpm at 2 Vpp, and increased their spinning speed non-linearly as the applied voltage rose. At 20 Vpp, the melan-a cells rotated at 400 rpm. In the same plot, the lymphocytes started to rotate at 2 Vpp at a speed of 7.4 rpm. The rotational speed was non-linearly proportional to the applied voltage. At 20 Vpp, the rotational speed of the cells was as fast as 363 rpm.

These results show that the rotational speed of specific cells depended on the AC voltage applied. The plots indicate a quadratic relationship between rotational speed and applied voltage, with the initial speed being zero when no voltage is applied. The relationship was found to be similar to that reported in traditional DEP setups [19], [23]–[24].

#### c) Rotational Speed vs. Frequency

**Figure 8:**
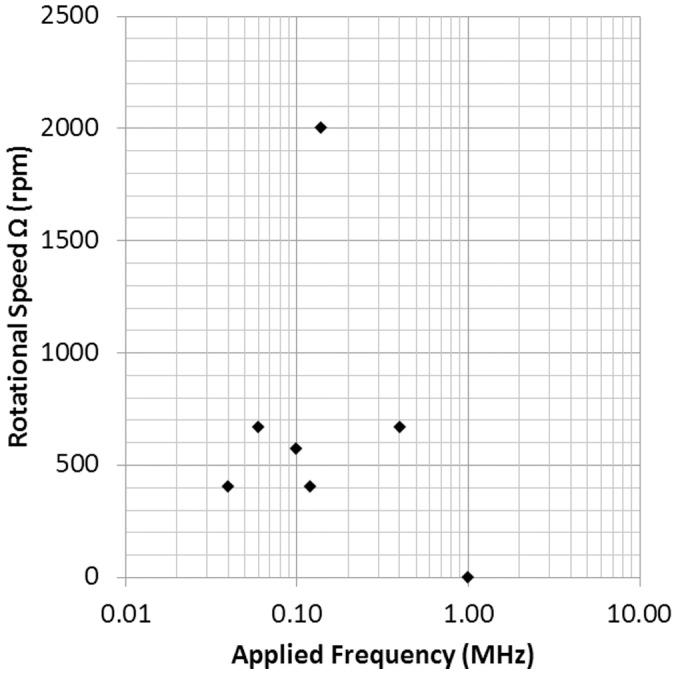
Rotational speed of the melan-a in 0.2 M sucrose solution versus applied frequency from 40 kHz to 1 MHz at 20 Vpp.

Figure 8 shows the rotational speed of melan-a cells versus applied frequency at 20 Vpp. The cells rotated at applied frequencies between 25 kHz and 800 kHz. At frequencies under 25 kHz, the melan-a cells moved swiftly into the image spot and eventually burst. If the applied frequency was too high, such as over 800 kHz, the cells neither moved toward nor were repelled by the spot, and they did not rotate in either the bright-field or the dark-field regions. The plot in Figure 8 shows that the fastest rotational speed of 2000 rpm was achieved when the applied frequency was 120 kHz.

**Figure 9:**
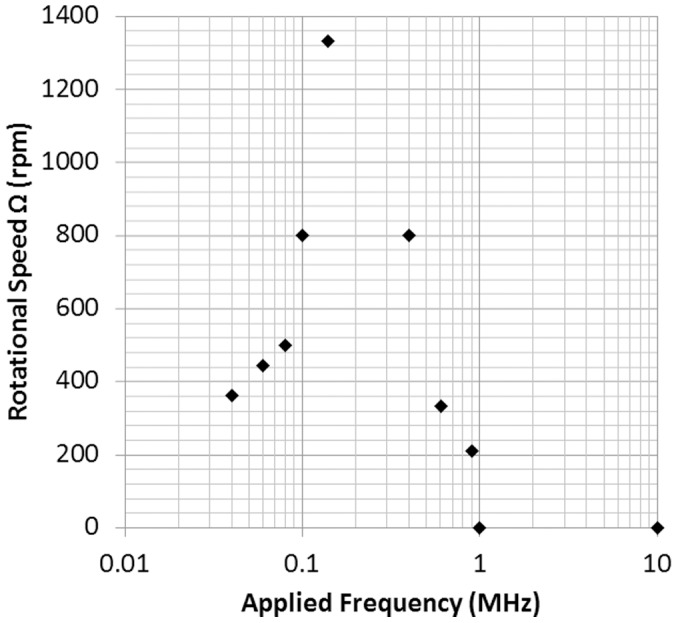
Rotational speed of the lymphocytes in 0.2 M sucrose solution versus applied frequency from 40 kHz to 1 MHz at 20 Vpp.

The lymphocytes also yielded results similar to those of the melan-a cells, as Figure 9 reveals. Frequencies over 1 MHz did not drive the cells to undergo translation and rotation. The cells started to rotate at a frequency of 800 kHz and reached the maximum speed of 1350 rpm at 120 kHz. The rotational speed of the cells decreased at frequencies from 120 kHz to 40 kHz. Frequencies below 20 kHz, which cause the heating effect, generated bubbles on the a-Si:H surface.

Figures 8 and 9 show that rotational speed is also associated with frequency. Both types of cells attained their highest rotational speed at a specific frequency and had no response in high frequency ranges. It is noticeable that the rotational speed of cells versus applied frequency is found to be very similar to the typical electrorotation spectrum [8], [25]. Hence, it is believed that the spinning effect is associated with DEP, but in an irrotational AC E-field.

#### d) Cell rotation inside the bright-field region

When melan-a cells entered the image spot, they did not rotate as they had in our previous research [21]. However, we observed the rotation of white blood cells and lymphocytes in the bright-field region. Table 3 shows the rotation analysis of WBCs, h-WBCs and s-WBCs. In the negative DEP region, all of the sample cells were in the dark-field region, with 95% of WBCs and h-WBCs undergoing rotation and 80% of s-WBCs rotating. The difference in the percentage of cells rotating indicates that the composition of the WBCs extracted from the blood vessels and spleens of mice were not the same. As the frequency increased from 50 kHz to 200 kHz, all of the WBCs and h-WBCs and 50% of the s-WBCs moved into the bright-field region under the influence of a positive DEP force. We observed that some cells in the image spot rotated. However, the proportion of each type of cell in the image spot differed, with 90% of WBCs, 90% of h-WBCs and 30% of S-WBCs rotating in the light image.

**Table 3:** Rotation behavior of white blood cell samples in the DEP field.

Applied Frequency (Hz) in 20 Vpp		<50 kHz		200 kHz<	
		Dark-field Region	Bright-field Region	Dark-field Region	Bright-field Region
**Induced rotation**	WBCs	95% rotate	N/A	95% rotate	90% rotate
	h-WBCs	95% rotate	N/A	95% rotate	90% rotate
	s-WBCs	80% rotate	N/A	90% rotate	30% rotate

Lymphocytes (b-/t- and NK-) also rotated in the bright-field region. Three lymphocytes were initially trapped in the image spot and one of them rotated at a constant speed. The rotating lymphocyte was isolated from the other cells and was moved to the cell-free area. A light spot with the same diameter and applied frequency and voltage was projected onto the rotating cell, but it did not rotate at all, even when the frequency was altered. The experiment was repeated and similar results were observed, indicating that the rotation of cells in the bright-field region was affected by the neighboring cells. Holzapfel et al. (1982) reported similar findings, specifically that two adjacent protoplast cells in the DEP field caused each other to rotate [24]. The author stated that cells can rotate only through cell-cell interaction or the local disturbance of the E-field. However, because cell-cell interaction can lead to a local disturbance in the E-field, the dipole moment of one cell interacts with the dipole moment of another cell to cause rotation.

Despite the above findings, a single cell can rotate alone in the dark-field region. The physical mechanism driving cell rotation in ODEP is still unclear. We hypothesize that rotation is due to the uneven distribution of mass within cells, such as the large nucleus incline in a lymphocyte or the uneven distribution of melanin in melan-a cells. When a dipole moment is created on the cell, it aligns the cell parallel to the E-field. If the center of gravity (C.G.) of the cell is not at its geometric center, the dipole moment may drive the cell to rotate continuously with sufficient DEP force. Hence, the physical properties of lymphocytes and melan-a cells may cause them to undergo self-rotation.

These explicit results show the induced rotation of the cells influenced by optically-induced DEP. With further experiments to explore how cells' rotational characteristics could be affected by their volume, morphology, organelles, and electrical properties, this observed self-rotation phenomenon could potentially be used to identify and separate different cell types. For example, we foresee that an automated system could be developed for the selection of cells in different cell-cycle phases. There are four phases in a cell cycle– G_1_ gap phase, synthesis (S) phase, G_2_ gap phase, and the mitosis (M) phase, and they are correlated to different dielectrics, such as changes in relative electrical permittivity [26], and physical properties, such as volume and size [8]. Therefore, based on our current results, it is entirely possible to segregate cells from the four cell-cycle phases by ODEP, and the segregation process could be automated by implementing computer-vision algorithms for real-time cell-rotation motion analysis and differentiation.

## Conclusion

In this paper, we describe the kinetics of WBCs, lymphocytes, macrophages and melan-a cells in an OEK device subjected to different AC voltages and frequencies.

The E-field generated in the OEK device was non-linear and irrotational. However, most of the WBCs and melan-a cells rotated. We hypothesize that the self-rotation of cells is due to the uneven distribution of their mass, such that once a dipole moment is created on a cell, it aligns itself to move parallel to the E-field. However, because a cell's center of mass is not located precisely at its center, the cell will continue to undergo rotation. This phenomenon is similar to that of an AC motor.

Much experimental work remains to be performed to derive a complete theoretical model for cell rotation in ODEP. Nevertheless, the cell rotation phenomenon observed here has great potential for use in constructing biomarkers for different cells. In addition to its application to cell identification, it may also be used to characterize cells in terms of their dielectric and mechanical properties, given that our results show rotational speed and translation motion to be inextricably linked to applied voltage and frequency.

## Supporting Information

Figure S1 **The ODEP system.** The actual ODEP system setup used to manipulate cells in our experiments.
(TIF)

Figure S2 **Fabrication procedure for the OEK device.**
(TIF)

Movie S1 **Induced rotation of melan-a and lymphocytes near the image spot.** A video recording showing the rotation of melan-a cells and lymphocytes around the 30 µm light spot in an OEK with the applied voltage of 20 Vpp and the applied frequency of 40 kHz.
(WMV)

Movie S2 **Induced rotation of white blood cells.** A video recording showing the rotation and manipulation of white blood cells in an OEK with the applied voltage of 20 Vpp and the applied frequency from 50 kHz to 200 kHz. Cells at 50 kHz were under negative DEP force and at 200 kHz were trapped in the optical image by positive DEP force. Cells were observed rotation in both bright and dark regions.
(WMV)

## References

[1] Bang H, Chung C, Kim J, Kim S, Chung S, et al (2006) Microfabricated fluorescence-activated cell sorter through hydrodynamic flow manipulation. Microsystem Technologies 12: 746–753.

[2] Davis JA, Inglis DW, Morton KJ, Lawrence DA, Huang LR, et al (2006) Deterministic hydrodynamics: Taking blood apart. PNAS 103: 14779–14784. 17001005 10.1073/pnas.0605967103 PMC1595428

[3] Roman G, Chen Y, Viberg P, Culbertson A, Culbertson CT (2007) Single-cell manipulation and analysis using microfluidic devices. Analytical and Bioanalytical Chemistry 387: 9–12. 16955261 10.1007/s00216-006-0670-4

[4] Wu Z, Hjort K, Wicher G, Fex Svenningsen A (2008) Microfluidic high viability neural cell separation using viscoelastically tuned hydrodynamic spreading. Biomed Microdevices 10: 631–638. 18461460 10.1007/s10544-008-9174-7

[5] Wu HW, Lin XZ, Hwang SM, Lee GB (2009) A microfluidic device for separation of amniotic fluid mesenchymal stem cells utilizing louver-array structures. Biomedical Microdevices 11: 1297–1307. 19731039 10.1007/s10544-009-9349-x

[6] Wu J, Ben Y, Battigelli D, Chang HC (2005) Long-range AC electroosmotic trapping and detection of bioparticles. Ind Eng Chem Res 44: 2815–2822.

[7] Minerick AR, Ostafin AE, Chang HC (2002) Electrokinetic transport of red blood cells in microcapillaries. Electrophoresis 23: 2165–2173. 12210220 10.1002/1522-2683(200207)23:14<2165::AID-ELPS2165>3.0.CO;2-#

[8] Kim U, Shu CW, Dane KY, Daugherty PS, Wang JYJ, et al (2007) Selection of mammalian cells based on their cell-cycle phase using dielectrophoresis. PNAS 104: 20708–20712. 18093921 10.1073/pnas.0708760104 PMC2410067

[9] Park K, Suk HJ, Akin D, Bashir R (2009) Dielectrophoresis-based cell manipulation using electrodes on a reusable printed circuit board. Lab on a Chip 9: 2224–2229. 19606300 10.1039/b904328d

[10] Chrimes A, Khoshmanesh K, Stoddart P, Kayani A, Mitchell A, et al (2012) Active control of silver nanoparticles spacing using dielectrophoresis for surface-enhanced Raman scattering',. Anal Chem 84: 4029–4035. 22468827 10.1021/ac203381n

[11] Vahey MD, Voldman J (2009) High-throughput cell and particle characterization using iso-dielectric separation. Anal Chem 81: 2446–2455. 19253950 10.1021/ac8019575 PMC2675787

[12] Chiou PY, Wong W, Liao JC, Wu MC (2004) Cell addressing and trapping using novel optoelectronic tweezers. 17th IEEE International Conference on Micro Electro Mechanical Systems 21–24.

[13] Liang W, Wang S, Qu Y, Dong Z, Lee GB, et al (2011) An equivalent electrical model for numerical analyses of ODEP manipulation. IEEE International Conference on Nano/Micro Engineered and Molecular Systems (NEMS) 825–830.

[14] Lin YH, Lee GB (2010) An integrated cell counting and continuous cell lysis device using optically induced electric field. Sensors and Actuators B: Chemical 145: 854–860.

[15] Lin YH, Lin WY, Lee GB (2009) Image-driven cell manipulation. IEEE Nanotechnology Magazine 3: 6–11.

[16] Liang YL, Huang YP, Lu YS, Hou MT, Yeh JA (2010) Cell rotation using optoelectronic tweezers. Biomicrofluidics 4: 043003. 10.1063/1.3496357 PMC3026025 21267435

[17] Morgan H, Green NG (2002) AC Electrokinetics: Colloids and nanoparticles. 1st ed. Research Studies Pr.

[18] Li M, Qu Y, Dong Z, Wang Y, Li WJ (2008) Limitations of Au particle nanoassembly using dielectrophoretic force: A parametric experimental and theoretical study. IEEE Transactions on Nanotechnology 477–479.

[19] Gimsa J, Marszalek P, Loewe U, Tsong TY (1991) Dielectrophoresis and electrorotation of neurospora slime and murine myeloma cells. Biophysical Journal 60: 749–760. 1835890 10.1016/S0006-3495(91)82109-9 PMC1260126

[20] Bennett DC, Cooper PJ, Hart IR (1987) A line of non-tumorigenic mouse melanocytes, syngeneic with the B16 melanoma and requiring a tumour promoter for growth. Int J Cancer 39 (3) 414–418. 3102392 10.1002/ijc.2910390324

[21] Chau LH, Ouyang M, Liang W, Lee GB, Li WJ, et al (2012) Inducing self-rotation of Melan-a cells by ODEP. IEEE International Conference on Nano/Micro Engineered and Molecular Systems 195–199.

[22] Loiko VA, Ruban GI, Gritsai OA, Berdnik VV, Goncharova NV (2007) Mononuclear cells morphology for cells discrimination by the angular structure of scattered light. 10th Conference on Electromagnetic and Light Scattering by Non-spherical Particles 105–108.

[23] Ouyang M, Zhang G, Li WJ, Liu WK (2011) Self-induced rotation of pigmented cells by dielectrophoretic force field. IEEE International Conference on Robotics and Biomimetics (ROBIO) 1397–1402.

[24] Holzapfel C, Vienken J, Zimmermann U (1982) Rotation of cells in an alternating electric field: Theory and experimental proof. Journal of Membrane Biology 67: 13–26. 7097755 10.1007/BF01868644

[25] Huang Y, Holzel R, Pethig R Wang XB (1992) Differences in the AC electrodynamics of viable and non-viable yeast cells determined through combined dielectrophoresis and electrorotation studies. Phys Med Biol 37: 1499. 1631195 10.1088/0031-9155/37/7/003

[26] Asami K, Takahashi K, Shirahige K (2000) Progression of cell cycle monitored by dielectric spectroscopy and flow-cytometric analysis of DNA content. Yeast 16: 1359–1363. 11054816 10.1002/1097-0061(200011)16:15<1359::AID-YEA631>3.0.CO;2-E

