# Supplementary figures and images for: Self-Rotation of Cells in an Irrotational AC E-Field in an Opto-Electrokinetics Chip
Source: PLoS One. 2013 Jan 8;8(1):e51577. doi: 10.1371/journal.pone.0051577 (PMC3540069; doi:10.1371/journal.pone.0051577)

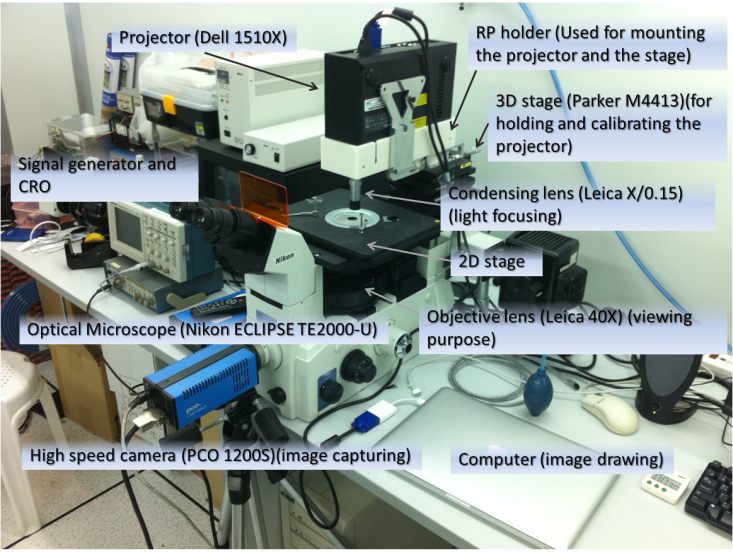

Supplement: Figure S1 — The ODEP system. The actual ODEP system setup used to manipulate cells in our experiments. (TIF) [file pone.0051577.s001.tif]

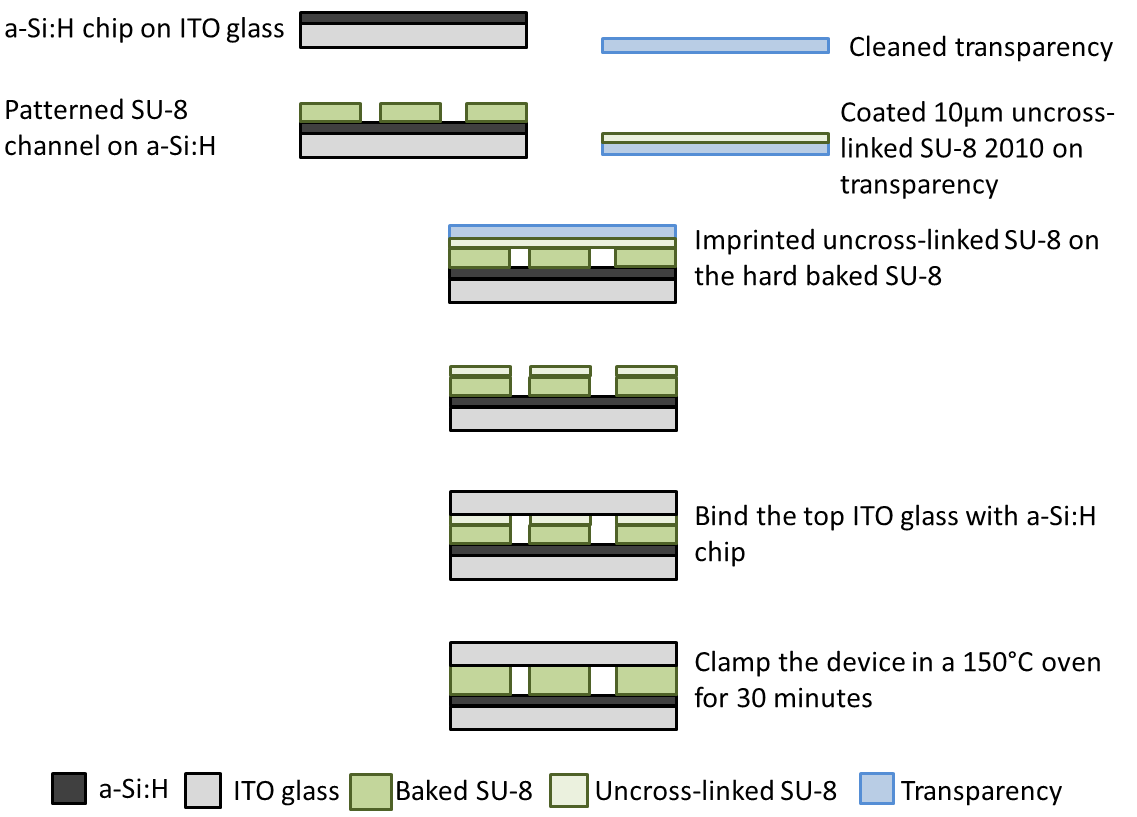

Supplement: Figure S2 — Fabrication procedure for the OEK device. (TIF) [file pone.0051577.s002.tif]
